# Resistance to sap-sucking insects in modern-day agriculture

**DOI:** 10.3389/fpls.2013.00222

**Published:** 2013-06-27

**Authors:** Arjen VanDoorn, Martin de Vos

**Affiliations:** ^1^Keygene NV, WageningenNetherlands; ^2^Department of Plant Physiology, Swammerdam Institute of Life Sciences, University of AmsterdamAmsterdam, Netherlands

**Keywords:** phloem-feeding insects, crop pests, breeding, genetically modified crops, natural insecticides

## Abstract

Plants and herbivores have co-evolved in their natural habitats for about 350 million years, but since the domestication of crops, plant resistance against insects has taken a different turn. With the onset of monoculture-driven modern agriculture, selective pressure on insects to overcome resistances has dramatically increased. Therefore plant breeders have resorted to high-tech tools to continuously create new insect-resistant crops. Efforts in the past 30 years have resulted in elucidation of mechanisms of many effective plant defenses against insect herbivores. Here, we critically appraise these efforts and – with a focus on sap-sucking insects – discuss how these findings have contributed to herbivore-resistant crops. Moreover, in this review we try to assess where future challenges and opportunities lay ahead. Of particular importance will be a mandatory reduction in systemic pesticide usage and thus a greater reliance on alternative methods, such as improved plant genetics for plant resistance to insect herbivores.

## EVOLUTIONARY PERSPECTIVE OF PLANT–INSECT INTERACTIONS

Around 350 m years ago, the first insects evolved to feed on plant material (Labandeira, 2007), after which plants evolved mechanisms to deter herbivores. These mechanisms include antibiosis, compounds toxic to insects, antixenosis, the deterrence of insect, physiological defensive properties, such as thorns and trichomes and tolerance (Smith and Clement, 2012). Insects on their part have evolved to detoxify or efficiently sequester these toxic metabolites. As early as 1888, Ernst Stahl elegantly demonstrated that extractable plant-based chemicals are responsible for defining host-specificity in plant–herbivore interactions (Stahl, 1888). It took until 1964 before the role of secondary metabolites in plants were again associated with insect host suitability, with Ehrlich and Raven (1964) in their landmark “plants and butterflies” paper. Here, the theory of co-evolution between plants and their herbivorous pests was laid out, and their paper was an important basis for subsequent plant–insect research.

## CROP DOMESTICATION AND MODERN AGRICULTURE

Over 4000 years, humans have been domesticating a large variety of crops; primarily selecting for “easy” traits, such as fruit size and yield. Evidently, in for example strawberries, the wild ancestors have much smaller berries and a completely different taste than the currently cultivated big, juicy, and often very sweet strawberry varieties (Aharoni et al., 2004). During this selection process and before global spread and subsequent outbreaks of pests and diseases, little or no attention was given to resistance beyond those required for locally occurring biotic and abiotic conditions. Therefore, many naturally occurring resistances have probably been lost (de-selected) during the cultivation of our current staple crops.

During the last century’s green revolution, crops were developed that are adapted to large-scale, high-input agriculture. This has driven an industrial-scale global agriculture and has, logically, resulted in industrial-sized seed production, for which a few suppliers in the EU and the USA provide seeds to a multitude of countries worldwide. The focus on high-input monocultures has advantages for industrial-sized agriculture, e.g., crops are easier to harvest, highly uniform, and produce predictably stable yields. However, such crop production also provides concerns and has drawbacks. Besides its high cost in energy input per unit arable land, one can also foresee that the use of these crop practices exert a tremendous selection pressure on pests and diseases, implying that resistances can easily be broken. In order to fight destructive herbivorous insects, humankind has heavily relied on the use of insecticides. However, in the last 15 years a large number of them, mostly systemic pesticides, have been banned because of their harmfulness toward consumers (e.g., parathion, dinitro-*o*-cresol), non-target organisms, or the environment [e.g., dichlorodiphenyltrichloroethane (DDT)]. More recently, neonicotinoids have come under fire because of harmful effects to non-target species such as bees and bumblebees (Henry et al., 2012; Whitehorn et al., 2012). Neonicotinoids are very effective pesticides as they are able to spread systemically throughout the plant, ensuring easy application and extending their usage in the formulations for seed coating. Overall, the EU and other countries worldwide have banned the use of many systemic pesticides^1^ because; (i) concerns about insecticide retention in food crops; (ii) effects on off-target organisms; (iii) broader negative impact on ecosystems, and (iv) higher risk of insecticide resistance in key insect pests.

## INSECT RESISTANCE IN MODERN-DAY BREEDING

With the current reduction in the range of pesticides that are available to farmers, efforts to find alternative methodologies for insect resistance have been on the rise. As a result, breeding for insect-resistant crops has received increased attention and many seed companies advertise their insect-resistant varieties. These insect resistance traits have come from a variety of sources, including plants and micro-organisms. For instance, broad resistance to Lepidoptera and Coleoptera is attained by the use of genetically modified (GM) plants, expressing a “Cry” toxin from *Bacillus thuringiensis *(Vaeck et al., 1987) in a number of important row crops, including corn, soybean, and cotton (Bohorova et al., 1996; Lambert et al., 1996; Stewart et al., 1996). Different Cry variants have been used in crops that exhibit differing spectra of efficacy against various groups of herbivorous insects, and are used widely in agriculture throughout the USA and other parts of the world^2^.Resistance or insensitivity to *Bt *in target insects has been observed in the laboratory (Meihls et al., 2012; Zhang et al., 2012) and the field (Gassmann et al., 2011). However, issues of insect resistance to *Bt *will be at least partly overcome in the latest generation *Bt*-crops, in which several Cry toxins, that do not show cross-resistance, are stacked or combined with other methodologies such as RNA interference (RNAi; Bhatia et al., 2012; Kumar et al., 2012; Tang et al., 2012) or the production of secondary metabolites. Evidently, the usage of *Bt*-crops has re-shaped the need for insecticide use, but has also allowed other, previously less economically important, insect pests to flourish. In particular, *Bt*-insensitive insects, such as aphids, whiteflies, and scale insects populations might increase in abundance. Hence, if GM strategies are to be used, these insect pests require other GM resistance strategies. GM approaches using plant-derived lectins, agglutinin, and protease inhibitors have been shown to provide high levels of resistance to aphids and other phloem-feeding insect species (Fitches et al., 2008; Alvarez-Alfageme et al., 2011; Carrillo et al., 2011). In addition, *in planta* expression of RNAi-vectors that target physiologically important insect transcripts for degradation, have been shown to result in crop protection against a number of insect pests, including phloem-feeding aphids (Price and Gatehouse, 2008; Upadhyay et al., 2011; Zha et al., 2011). Although potentially effective, none of these GM methodologies have been commercially marketed. A variety of reasons can underlie their lack of success on the market, these include (i) high risk of limited durability, particularly if less than 99% mortality is achieved; (ii) potential negative effects on non-target insects, ecosystems, or consumers; (iii) narrow target-specificity, i.e., high cost of deregulation of a GM does not pay off compared to the reduction in yield loss resulting from an economically minor pest or a niche market crop.

Hence, there is a strong incentive to develop alternative strategies against these pests. In that respect, combined approaches seem particularly attractive. For instance, the use of (non-GM) genetic crop resistance, combined with biological control using predatory insects or practical solutions that limit the build-up of high population densities of herbivorous pests will likely result in effective pest control.

## BENEFITTING FROM NATURAL VARIATION

An alternative to transgenic approaches is the use of wild relatives of crop plants, searching for desirable traits and then crossing those into the elite cultivars. This traditional way of plant breeding has been made substantially easier with the availability of novel sequence-based molecular approaches. For instance, genome-wide coverage of single nucleotide polymorphisms (SNPs; or other molecular markers) between wild and cultivated species are easily obtained and make marker assisted selection or marker assisted breeding for traits of interest feasible in many crops. Moreover, genome-wide association studies to identify SNPs linked to traits of interest and the subsequent use of novel breeding schemes (breeding by design) will further revolutionize crop breeding for insect resistance. All these methodologies are advanced by whole genome sequencing of crop plants, e.g., maize, rice, wheat, but also vegetable crops such as tomato, lettuce, and cabbage (Goff et al., 2002; Schnable et al., 2009; Brenchley et al., 2012; Sato et al., 2012), and re-sequencing of wild germplasm. However, a challenge remains when traits are polygenic, and the individual components have subtle effect. Moreover, the genetic background of elite cultivars might interfere with traits from wild relatives. There is a clear need to bridge the current gap in the understanding of these technological advances between bio-informaticians, bio-statisticians, entomologists, plant pathologists and (pre-) breeders. It is often overlooked that only their collective efforts will ensure important breakthroughs in pest and disease resistance in crops.

## R-GENE-MEDIATED RESISTANCE TO INSECT PESTS

Although some resistances are effective against a broad range of pest species, most are highly herbivore-specific reactions. Exploitation of natural resistances, often found in wild relatives that are interbreedable with our current crops, is well-suited to combat pest species that consume a specific plant organ or tissue (e.g., aphids, whiteflies, and other phloem-feeding insects).

R-gene-based resistance relies on a “gene-for-gene” interaction, where a compound secreted by the insect is specifically recognized by the plant, thus enabling the plant to initiate a defense response.

Whereas R-gene-mediated resistance has not been established for tissue chewing insects (i.e., Lepidoptera and Coleoptera), several examples of strong monogenic natural resistance to phloem-feeding pests have been reported in literature. Only a few of these dominant R-genes – that provide resistance against phloem-feeders – have been cloned (e.g., Mi-1.2, VAT, and BPH16) and many more are extensively used in agricultural settings through the use of marker assisted breeding (for a recent review, see Broekgaarden et al., 2011; **Table 1**).

**Table 1:** Overview of R-genes mediating insect resistance (adapted from Broekgaarden et al., 2011, with permission).

Plant species	Gene	Insect	Resistance broken	Reference
*Triticum aestivum*	*H genes*	*Mayetiola destructor*	yes	Wang et al.(2006);Yu et al. (2009), and Harris et al. (2012)
	*Dn* genes	*Diuraphis noxia*	yes	Liu et al. (2005),Peng et al. (2009), and Tolmay et al. (2007)
*Oryza sativa*	*Bph* genes	*Nilaparvata lugens*	yes	Du et al. (2009),Qiu et al. (2010) and Peñalver Cruz et al. (2011)
	*Gm genes*	*Gall midge*		Himabindu et al. (2010), and Kumar et al. (2009)
*Solanum lycopersicum*	*Mi-1.2*	*Macrosiphum euphorbiae, Bemisia tabaci*	yes	Rossi et al. (1998),Nombela et al. (2003),and Goggin et al. (2001)
*Cucumis melo*	*Vat*	*Aphis gossypii*	yes	Klingler et al. (2001) and Lombaert et al. (2009)
*Medicago truncatula*	*AIN*	*Acyrthosiphon kondoi*	yes	Klingler et al. (2009) and Humphries et al. (2010)
*Glycine max*	*Rag genes*	*Aphis glycines*	yes	Li et al. (2007); Zhang et al. (2009,2010) and Kim et al. (2008)

Therefore, it is tempting to draw a general conclusion about R-gene-mediated insect resistances found in nature: only those pests, such as phloem-feeding insects, that require an intimate relationship with their host plant to successfully colonize are likely to be contained using R-gene-mediated defenses.

Interestingly, even in crops where the R-gene is cloned and characterized, the mode-of-action of these resistances is unclear. It should involve attacker recognition and down-stream signal transduction leading to an effective defense response that results in the inability of phloem-feeding insects to establish prolonged feeding.

Similarly to plant–pathogen interactions, the cloned insect resistance genes are members of the family of nucleotide-binding, leucine-rich repeat (NBS-LRR). Therefore, in analogy with pathogen recognition, it is expected that recognition of insect herbivores by NBS-LRR proteins takes place through direct or indirect binding of insect effector molecules (Dodds and Rathjen, 2010). Effector molecules of phloem-feeding insects are thought to be secreted into the host plant during probing (testing phase) or subsequent prolonged feeding (ingestion of phloem sap; Will et al., 2007; Moreno et al., 2011). Although several candidate effector molecules, e.g., secreted from the salivary glands of aphids, have been identified (Ramsey et al., 2007; Harmel et al., 2008; Carolan et al., 2009; Bos et al., 2010; Rodriguez and Bos, 2013), none of these have been associated with the binding by R-genes directly, or to known so-called virulence targets “guarded” by R-genes. It is expected that this field of research will take an enormous flight and shows a promise for plant breeding for insect-resistant crops.

The *Mi-1.2* gene in tomato, arguably most researched, is extensively used for control of root-knot nematodes [*Meloidogyne* species (Milligan et al., 1998; de Vos et al., 2008)], but also is effective against some clones of the tomato–potato aphid (*Macrosiphum euphorbiae*; Rossi et al., 1998), whiteflies (*Bemisia tabaci*; Nombela et al., 2003), and the potato psyllid (Casteel et al., 2006)]. This broad effectiveness of the Mi gene toward several tomato phloem-feeding pests is striking and suggests recognition of several species-specific effector molecules. As an alternative – and more likely – hypothesis one would expect these insect species use a similar gateway, guarded by Mi-1.2, to successfully colonize tomato. To date, no such effector from either of the insect, nor *Meloidogyne* species, has been identified that causes the hypersensitive response in *Mi1.2* tomato plants. Mi-mediated resistance to root-knot nematodes in characterized by a local hypersensitive response that takes place within 24 h upon feeding by *Meloidogyne* species. The Mi-mediated response to aphids is clone-specific and requires common signaling components characterized for pathogen defenses (Bhattarai et al., 2007,^2010^; Atamian et al., 2012).

Over the past decades plant breeding companies have exploited natural variation for dominant monogenic insect resistance genes. The genes described above are extensively used in horticulture. Other dominant loci, such as those required for resistance against wheat against the Russian wheat aphid or Hessian flies have been extensively used in agricultural settings. The large-scale usage of these dominant loci has resulted in newly arisen insect populations (virulent biotypes). For example, aphid biotypes of *Nasonovia ribisnigri* have been identified in Europe that are able to feed from cultivated lettuce carrying a dominant monogenic resistance introgressed from *Lactuca virosa *(Thabuis et al., 2011)*.* Other examples, include virulent biotypes of the Russian wheat aphid that break through Dn resistance in wheat (Haley et al., 2004).

Pyramiding of R-genes (similar to *Bt*-approaches), where more than one resistance trait is stacked, can possibly prevent, or at least delay, the formation of new insect biotypes that can evolve to feed on resistant crops and this strategy can contribute to increased durability of these resistances. This may be a more responsible use of the currently limited set of available resistance traits. Ultimately, the decision to pyramid resistance genes will depend on several, often economic, factors, including (i) the availability of natural germplasm; (ii) the current and future economic threat of a pest; (iii) the population characteristics of the pest and its ability to evolve counter measures that lead to insensitivity; (iv) the time-to-market for the crop at hand, and (v) the level of resistance in current (competitive) commercial varieties.

## METABOLITE-MEDIATED RESISTANCE

As described above, R-gene-based defense can render strong species-specific resistance to a limited set of herbivores, but is certainly not effective against all herbivores. The constitutive or induced production of secondary metabolites can provide an alternative resistance strategy. These compounds, which may be specific for the plant genus or family, often accumulate in leaf tissue where they occur in specialized structures on the plant’s surface or are compartmentalized within the host cell.

**FIGURE 1:**
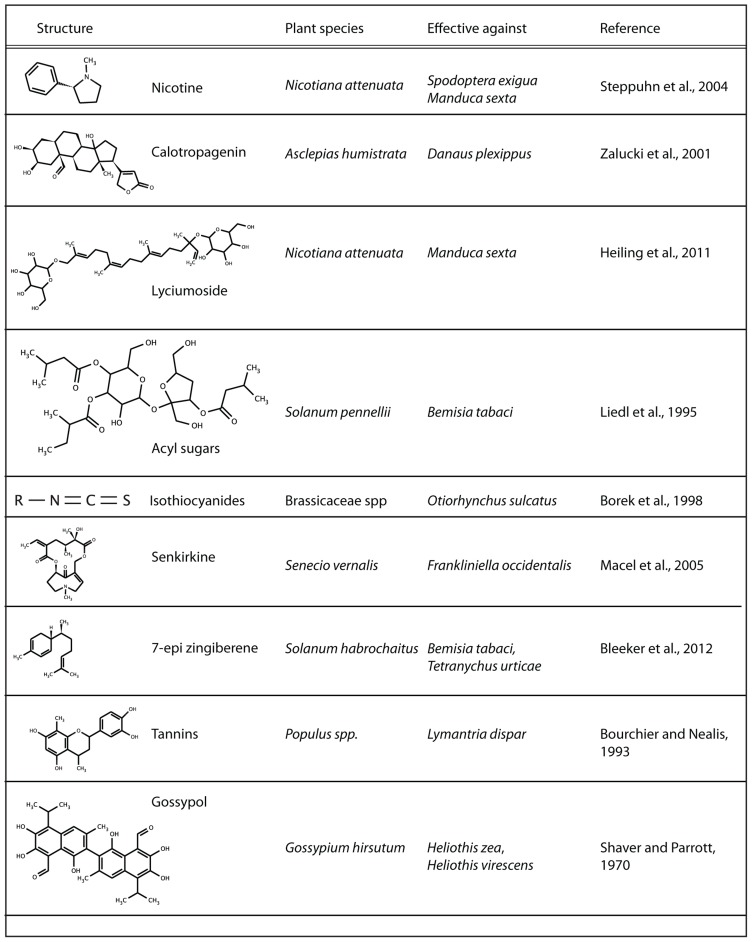
**Overview of insecticidal secondary metabolites and their plant origin**.

There is an incredible natural diversity of compounds present in plants (**Figure 1**). Whereas some of the biosynthetic pathways are restricted to a certain family, others are spread throughout the plant kingdom. Examples of specialized plant metabolites are glucosinolates in *brassicaceae*, from which toxic and anti-feedant compounds are enzymatically formed as soon as the cells are ruptured by herbivore feeding (Lüthy and Matile, 1984). Moreover, a wide variety of alkaloids have been identified, such as the neurotoxin nicotine in *Nicotiana attenuata* (Steppuhn et al., 2004), saponin glycoalkaloids in tomato (Chan and Tam, 1985) and pyrrolizidine alkaloids in chrysanthemum (Macel et al., 2005) that are related to resistance to generalist insect pests.

On the contrary, compounds such as terpenes occur ubiquitously throughout the plant world, and are synthesized through common pathways present in most plants. However, there is also immense structural variation in these terpenes themselves, with an estimated 30,000 different structures occurring in plants (Connolly and Hill, 1991; Degenhardt et al., 2009b; Pichersky and Lewinsohn, 2011). Small changes in the final biosynthetic enzymes (terpene synthases), the availability of substrates and the biosynthetic conditions in the cells play a defining role in determining which terpenes are produced (Degenhardt et al., 2009a). This has provided plants with an enormous evolutionary flexibility to fine-tune the chemical responses to herbivory.

Because many terpenes are volatile, and many plants induce their production when attacked by herbivores, they provide an opportunity for predators to locate herbivore-infested plants, and serve a role as semiochemicals (information-conveying chemicals; Turlings et al., 1990; Kessler and Baldwin, 2001). A very diverse set of terpenoids has been suggested to play a role in indirect defense, such as bergamotene in wild tobacco, and a blend of mono- and sesquiterpens in tomato (Kessler and Baldwin, 2001; Kant et al., 2004).

Terpenes have also been shown to act as direct toxins to a suite of insects and pathogens [e.g., 7-epi-zingiberene against whiteflies (Bleeker et al., 2011), resins against bark beetles in confiners (Phillips and Croteau, 1999), and terpenoid lactones against Colorado potato beetles (Szczepanik et al., 2005)], but are at high concentrations also toxic to the plant itself (Aharoni et al., 2003). Therefore, plants sequester and compartmentalize terpenes, transport them to the leaf surface, or produce and store terpenoids in trichomes. The latter allows a terpene coating toward the outside environment of the plant without the need to adapt to high intercellular concentrations of these compounds.

A major pest in commercial tomato cultivation is the whitefly *Bemisia tabaci*, mainly because it is a vector for *begomo* viruses, causing substantial losses in commercial vegetable cultivation (Navas-Castillo et al. , 2011). Although some promising sources of resistance have been identified (Firdaus et al., 2013), to date no R-gene-based resistance has been identified for *Bemisia tabaci*, a highly polyphagous phloem-feeding insect with a host-range spanning over 100 plant species (Mound and Halsey, 1978). Although a whitefly population can quickly reach enormous numbers, their direct impact on crop yield is limited. In contrast, indirect damage from whitefly vectored viruses is a major threat to crop production. To prevent virus vectoring by sap-sucking pests one should ideally rely on a complete avoidance response of the insect toward the host plant. Volatile-mediated repellency of whiteflies might just provide such an opportunity in tomato, where tomato yellow leaf curl virus (TYLCV) is a major agricultural disease transmitted by *Bemisia tabaci*.

By screening a number of wild tomato plants for repellence against whiteflies, Bleeker et al. (2011) found that *Solanum habrochaites* showed strong repellency to whiteflies. Subsequently, the repellency was shown to be mediated by a sesquiterpene, namely 7-epi-zingiberene (Bleeker et al., 2011). 7-epi-zingiberene is exclusively produced in the glandular trichomes of *S. habrochaites *(Bleeker et al., 2012). In the offspring of interspecific crosses between *S. habrochaites* and cultivated tomato (*S. lycoperiscum*) were made, the F2 plants showed a strong correlation between 7-epi-zingiberene production and whitefly resistance. Surprisingly, this compound did not only confer resistance against whiteflies, but also against other herbivorous pests with entirely different modes of feeding, these include single-cell feeders (the spider mite *T. urticae*) and caterpillars (*Manduca sexta*; Bleeker et al., 2012). The above-described approach is very promising for multiple (vegetable) crop species. The repellent and toxic effects of such compounds produced at the plant–environment interface (e.g., in glandular trichomes) directly functions as an alarm bell that signals “inedible” to approaching herbivorous pests, but will be particularly important in fighting off virus vectoring insect species.

## WHAT CHALLENGES ARE AHEAD

Preventing pre- and post-harvest damage caused by insects is a very challenging, but economically important, issue for plant breeders. Particularly, the proposed and partly implemented reductions in the use of systemic pesticides will further increase the need of genetic host resistance in the near future. GM approaches have been extremely successful in controlling some insect species, but their implementation, particularly in the EU, face heavy political opposition. Moreover, due to the de-regulatory process, GM introduction is expensive, thereby making it less feasible for the smaller vegetable crop markets, which are often locally tailored and also diversified to achieve specific consumer traits.

In order to have a chance against insect species that have multiple generations in a year, it is of crucial importance to widen our understanding of resistances in wild relatives of our current crops against insect herbivores. This will be an essential responsibility for plant pathologists, entomologists, breeders, and the entire research community. It has been estimated that for crops such as tomato, there is a multitude of gene-information “buried” in wild species that can be crossed with elite varieties. This genetic reservoir represents a largely untapped treasure for new or improved traits that could make our current crops significantly more productive. Because every day more genomic sequences are becoming available, this enables a quicker trait-to-gene path, thus providing a good academic opportunity to look beyond model plants and provide an insight in unique traits of wild species. Large efforts will need to be made to understand what genes are underlying the traits of future importance.

## Conflict of Interest Statement

The authors declare that the research was conducted in the absence of any commercial or financial relationships that could be construed as a potential conflict of interest.

## Footnotes

1 EU directive 2009/128/EC for sustainable use of pesticides in plant protection.

2 ISAAA Brief 43-2011. Global Status of Commercialized Biotech/GM Crops in 2011. Or summary at
